# Supplementary material for: Z-flipon variants reveal the many roles of Z-DNA and Z-RNA in health and disease
Source: Life Sci Alliance. 2023 May 10;6(7):e202301962. doi: 10.26508/lsa.202301962 (PMC10172764; doi:10.26508/lsa.202301962)
Supplement: Supplemental Data 1. — Overlap of Z-flipons with ENCODE cancidate cis regulatory regions. [file LSA-2023-01962_Supplemental_Data_1.docx]

Supplemental Materials

Learning Z-DNA sequences from attention maps

Transformer architecture allows interpretation of important features by analysis of attention maps. Results can be interpreted according to the difference in the expected frequency of k-mers in the input sequence versus their rank in the output and compared to the frequency in the genome or in the genomic region of interest. This approach is helpful for assessing ChIP-seq data, as a priori, the distribution of Z-HUNT3 predicted Z-flipons in the genome is highly biased towards promoters. Many sequences associated with promoters, such as TATA boxes or GC rich segments will have high frequencies in the pull-downs independently of their ability to flip to Z-DNA.

The distributions of 6-mers according to their rank in the attention map are given in Table 2. When the model is learning it pays attention not only to the k-mers inside Z-DNA regions but also to the k-mers in the flanking regions. For example, according to attention ranking k-mer GGGGAA is the 7th most frequent that the model uses to define Z-DNA, however this k-mer is the 40th according to the frequency of occurrence inside Z-DNA regions. Also, k-mers GGGGAA, CAGGGA, TGGGGA, GGGGGA, AGGGAG, GGGAGC are rarely at the site of Z-DNA nucleation, they likely can propagate the flip to Z-DNA once it is initiated. In the model, they appear important for Z-DNA prediction in the nearby sites where alternating pyrimidine/purine sequences may be the first segments to flip conformation.

To investigate further how Z-DNABERT recognizes GT and CA repeats we selected regions from Kouzine et al. human dataset. These repeats are located within 10 bp of each other. Summary attention heatmaps (sum of attention weights from all 12 heads for each position) for various regions are depicted in Fig S1.

Z-DNABERT cross-species predictions and other applications

We tested how well Z-DNABERT model trained on one genome can predict Z-DNA regions in another genome. Table 3 shows the results of the model performance that was trained on human and then applied to the mouse genome using Kouzine et al. data sets. Performance metrics remain high.

We have provided a readme file for the resource at:

https://github.com/mitiau/Z-DNABERT/blob/main/README.md

and a Z-DNA prediction tool published at

https://colab.research.google.com/github/mitiau/Z-DNABERT/blob/main/ZDNA-prediction.ipynb

A user can input a sequence of interest into our pretrained model to identify Z-flipons with a high level of confidence. As mentioned in the test, we have been able to identify flipons in RNAs immunoprecipitated with the anti-ZNA antibody Z22 (Zhang T et al, 2022).
